# Action 3:30R: Results of a Cluster Randomised Feasibility Study of a Revised Teaching Assistant-Led Extracurricular Physical Activity Intervention for 8 to 10 Year Olds

**DOI:** 10.3390/ijerph16010131

**Published:** 2019-01-06

**Authors:** Russell Jago, Byron Tibbitts, Emily Sanderson, Emma L. Bird, Alice Porter, Chris Metcalfe, Jane E. Powell, Darren Gillett, Simon J. Sebire

**Affiliations:** 1 Centre for Exercise, Nutrition & Health Sciences, School for Policy Studies, University of Bristol, Bristol BS8 1TZ, UK; b.tibbitts@bristol.ac.uk (B.T.); alice.porter@bristol.ac.uk (A.P.); simon.sebire@bristol.ac.uk (S.J.S.); 2 The National Institute for Health Research Collaboration for Leadership, Applied Health Research and Care West (NIHR CLAHRC West), University Hospitals Bristol NHS Foundation Trust, Bristol BS1 2NT, UK; 3 Bristol Randomised Trials Collaboration, Bristol Trials Centre, University of Bristol, Bristol BS8 2PS, UK; emily.sanderson@bristol.ac.uk (E.S.); chris.metcalfe@bristol.ac.uk (C.M.); 4 Centre for Public Health and Wellbeing, University of the West of England, Bristol BS16 1QY, UK; emma.bird@uwe.ac.uk (E.L.B.); Jane.Powell@uwe.ac.uk (J.E.P.); 5 Bristol City Council–Sport & Healthy Lifestyles Team, Healthy Lifestyles Healthy Place, Public Health City Hall, Bristol BS3 9FS, UK; darren.gillett@bristol.gov.uk

**Keywords:** Keywords: physical activity, children, teaching assistants, intervention, feasibility, after-school

## Abstract

Many children are not sufficiently physically active. We conducted a cluster-randomised feasibility trial of a revised after-school physical activity (PA) programme delivered by trained teaching assistants (TAs) to assess the potential evidence of promise for increasing moderate-to-vigorous physical activity (MVPA). Participants (*n* = 335) aged 8–10 years were recruited from 12 primary schools in South West England. Six schools were randomised to receive the intervention and six acted as non-intervention controls. In intervention schools, TAs were trained to deliver an after-school programme for 15 weeks. The difference in mean accelerometer-assessed MVPA between intervention and control schools was assessed at follow-up (T1). The cost of programme delivery was estimated. Two schools did not deliver the intervention, meaning four intervention and six control schools were analysed at T1. There was no evidence for a difference in MVPA at T1 between intervention and control groups. Programme delivery cost was estimated at £2.06 per pupil per session. Existing provision in the 12 schools cost £5.91 per pupil per session. Action 3:30 was feasible to deliver and considerably cheaper than existing after-school provision. No difference in weekday MVPA was observed at T1 between the two groups, thus progression to a full trial is not warranted.

## 1. Introduction

Physical activity is associated with lower levels of cardiometabolic risk factors [1], lower risk of obesity [2] and improved psychological well-being in children and adolescents [3,4] and adults [2]. Nationally representative data from the UK Millennium Cohort showed that only half of 8–9 year old’s in the UK engaged in the recommend 60 min of moderate to vigorous intensity physical activity (MVPA) per day [5]. Physical activity levels decline as children move through primary school [6] and as such there is a need to find ways to maintain physical activity during primary school.

The school setting provides opportunities to recruit large numbers of children simultaneously, making it an opportune setting for interventions to increase children’s physical activity levels [7]. Systematic review evidence indicates that physical activity interventions during curriculum time have had limited success [8]. Those intervention studies conducted during curriculum time that found an effect were mainly studies of low methodological quality or robustness. Limitations of these studies included inadequate adjustment for potential confounders, not adjusting for the clustering of the data, and the use of self-report measures of physical activity [8]. Alternative intervention approaches evaluated using robust study design and methods are therefore needed.

The time immediately after school is discretionary time for most children [9] and could be used to promote physical activity [10]. A study with 952 primary school children found that children who are inactive in the period straight after school (between 3 p.m. and 6 p.m.) are less likely to meet PA guidelines [9]. Therefore, after-school programmes that maximise the opportunities for physical activity could be a means of effectively increasing physical activity levels of children at primary school [7,11]. Current after-school provision in UK primary schools is dominated by team sports and external practitioners, such as football coaches [12]. This is costly to schools and parents and is likely to mainly appeal to children who are already engaged with structured physical activity.

In 2017 the UK government doubled the primary school PE and Sport Premium to £320 million and recommended that funds be used to make sustainable improvements to the quality of physical activity opportunities offered across the school day, including after school [6]. A stated key indicator for schools which is tied to the increased funding is evidence of increased confidence, knowledge and skills of staff in teaching PE and sport [6]. Offering training and resources to existing staff, such as teaching assistants, to equip them to deliver active after-school programmes aligns with government recommendations for schools and could be a low-cost means of helping children to be more active.

We have previously developed a programme, called Action 3:30, which trained teaching assistants (TA) to deliver a physical activity club after-school. Action 3:30 was underpinned by Self-Determination Theory (SDT) [13]. The evaluation of the original Action 3:30 programme indicated that training TAs to deliver a physical activity club in the after-school period holds promise [11]. However, there was a need to increase the recruitment of less active pupils, increase the effect on girls and boost attendance. The program content was revised to address these issues and as such there is a need to evaluate the revised programme and particularly whether it yields an increase in the physical activity of both boys and girls and results in higher attendance levels. Furthermore, there is also a need to examine the potential utility of new approaches to recruiting low active children and whether additional children could be added to the program while the program is still running by adopting a rolling-recruitment approach.

The aim of this study is to test the feasibility and potential for effectiveness of the revised version of Action 3:30. In this paper we report on the recruitment process, feasibility of conducting a definitive trial, potential efficacy of the trial and the likely cost of the intervention. Finally, we assess whether progression criteria for conducting a definitive trial were met (see Methods).

## 2. Methods

The full methodology and study protocol are described in detail elsewhere [14] and outlined below.

### 2.1. Participants

Twelve state-funded primary schools from South Gloucestershire (*n* = 8 schools) and North Somerset (*n* = 4 schools) in Southwest England were recruited to the study. The target population for this study were pupils in Year 4 and 5 (aged 8 to 10 years) at the time of the intervention. We aimed to recruit up to 30 children per school.

### 2.2. Recruitment

Findings from the previous Action 3:30 feasibility study indicated that more work was needed to recruit low-active children and to appeal to girls [11]. In this revised feasibility trial two recruitment methods were tested to examine which of the two approaches was most effective. Recruitment method A (standard) consisted of a project briefing to all eligible pupils in the school. Recruitment method B (enhanced) was a briefing plus a 20–30 minute taster where pupils experienced a sample Action 3:30R session [14]. Recruitment took place in two local authority areas. Four schools from South Gloucestershire and two from North Somerset were randomly selected to receive the taster sessions.

### 2.3. Study Design

This feasibility study used a cluster randomised trial design. Data were collected at two time points: baseline (T0), and then during the final six intervention sessions (T1). To compare the activity levels of children who consented to join the study with the overall sample of eligible children there was an additional (pre-baseline) data collection prior to T0.

Schools were randomly assigned to intervention (6 schools) or control (6 schools) arms after baseline data had been collected. Allocation was stratified by Local Authority and recruitment method: standard or enhanced. Random allocation and statistical analyses were conducted using Stata by an independent member of the Bristol Randomised Trial Collaboration who was blinded to school identity. Written parental informed consent was obtained for all participants for the T0 and T1 phases of the study. An additional, “opt-out” consent process was used for the pre-baseline survey phase of the project as outline in Section 2.5.1 below. The study received ethical approval from the School for Policy Studies Research Ethics Committee at the University of Bristol (ref: SPSREC16-17.B2).

### 2.4. Intervention

The Action 3:30 intervention has been described in detail elsewhere [14]. Briefly, two TAs from each of the intervention schools were required to attend five days (25 h) of training. The Action 3:30 after-school clubs were scheduled to run twice per week for 15 weeks in each school and last 60 min per session. Sessions were designed to promote maximal participation, skill development, cooperation, problem solving, physical activity and choice. The sessions began with fun warm-up activities, and the moved through a series of small sided games and activities with a focus on fun and participation while also improving fundamental movement skills such as running, catching, throwing and use of space in invasion games. TAs were trained to promote, and foster aspects of motivation drawn from SDT within the club with a focus on creating a club climate which supported autonomy, relatedness and competence. TAs completed a log book to indicate whether sessions were delivered fully, partially or not at all and a register of attendance.

#### Changes from the Previous Intervention

Based on findings from the outcome [11] and process evaluations [15] of the original Action 3:30 study, the intervention was refined to improve appeal to girls, provide example activity demonstration videos and help the Teaching Assistants to manage disruptive behavior.

### 2.5. Measures

#### 2.5.1. Phase 1: Pre-Baseline Activity Survey

In order to compare those pupils who consented to join the study with all remaining eligible pupils, all children in the 12 study schools were asked to complete the Physical Activity Questionnaire for Older Children (PAQ-C) [16] prior to the main study commencing when they were in years 3 and 4. The mean PAQ-C is scored on a 1-5 scale with higher scores indicating higher activity levels. To assess whether the final sample included children who were representative of the possible sample, in four South Gloucestershire schools, all children were also asked to wear an accelerometer for 7 days. The activity levels of the participants who consented to join the study could then be compared to those who did not join the study. For this phase of the study parents were notified of the study goals and given the opportunity to opt their child out of the study as part of a protocol that was agreed with the ethics committee. If no opt-out form was received the students completed the PAQ-C and, if in one of the four schools selected for the additional phase, asked to wear an accelerometer. Finally, to mirror the processes that are common in most UK primary schools we had an additional phase half way through the intervention period in which we invited pupils who had not consented to join at T0 to join the study and provide follow-up data at the T1 assessment along will all other pupils. A total of 18 additional pupils were recruited via this method. As this provides limited additional information these data have not been reported here.

#### 2.5.2. Baseline (T0) and Follow Up (T1)

T0 was conducted when the target pupils were at the end of years 3 and 4, approximately 4 months before the start of the intervention. This was done to facilitate to enable the intervention schools to be notified of allocation after baseline data had been completed, while also allowing time for TAs to attend training. T1 was conducted in the last six weeks of the intervention period when the children were in years 4 and 5 respectively and therefore provided an indication of the levels of physical activity while the program was still running. Pupils wore ActiGraph wGT3X-BT accelerometers (Actigraph LLC, GL) for seven consecutive days to measure physical activity at both time points. Pupils were included in analysis if they provided ≥ 3 valid days (500 min) of data. Minutes of moderate-vigorous physical activity (MVPA) were estimated using the Evenson cut-point [17]. Total physical activity was derived from counts per minute (cpm) and sedentary time was derived based on a cut-point of less than 100 cpm. Height and weight were measured by a trained researcher and Body mass index (BMI) was calculated and converted to an age and sex specific standard z-score [18].

Pupils also completed a questionnaire assessing PA motivation and activity-based perceptions of autonomy, relatedness and competence needs satisfaction [19]. PA motivation was assessed using a 5-point Likert scale ranging from zero *(not true for me)* to four *(very true for me)*. Three questionnaire items were used to assess each of four types of motivation (intrinsic, identified, introjected and external) with the mean of all three items calculated for each type of motivation. Composite scores for autonomous (mean of intrinsic and identified) and controlled (mean of introjected and external) motivation were then calculated. Autonomy, competence and relatedness needs satisfaction were each assessed using a 6-item Likert scale, ranging from one *(not like me at all)* to six *(really like me)*. Parents were asked to complete a questionnaire to collect demographic data (T0 only), travel mode to and from school and pupils’ current participation in after-school clubs. Demographic information included ethnicity and parental education level.

Health-related quality of life (HRQoL) was assessed using the KIDSCREEN-10 [20] and Child Health Utility 9D (CHU9D) [21] questionnaires, which informed part of the economic evaluation. The KIDSCREEN-10 instrument included 12 items, 11 of which were given on a 5-point Likert scale and one given with a yes or no response option (item relating to whether pupils have a long-term disability or illness). KIDSCREEN-10 scores were converted into T-scores as per the KIDSCREEN guidelines [22]. T-scores have a mean of 50 and a standard deviation of 10, with higher values indicating higher HRQoL. The CHU9D instrument included nine items, each with five response options. Responses were converted to utility scores using CHU9D SPSS Syntax [23]. Utility scores range from 0-1 on a scale, where from 1.0 is defined as perfect health and 0.0 is a state considered equivalent to being dead [23,24]. These scores provide an indication of the HRQoL of a child; the higher the CHU9D score, the better the health-related quality of life.

### 2.6. School and Pupil Appreciation

Intervention schools received £200 to buy equipment to deliver the club and were reimbursed to cover the cost of two hours per week for two TAs to deliver the club. As recompense for the additional work involved in facilitating data collection control schools received a one-off payment of £300. Pupils received a small gift at each data collection in recognition of their contribution to the study.

### 2.7. Statistical Analysis

Summary statistics were presented to describe control and intervention arms at T0 and T1 for demographics (T0 only), accelerometer, psychosocial and health-related quality of life variables. Data provision rates for accelerometer (valid, invalid, missing) and questionnaire data (complete, partially complete, missing) were recorded at T0 and T1.

To estimate the potential intervention effect, an intention-to-treat (ITT) analysis using a multivariable mixed effects linear regression that took account of the clustering of pupils in schools was conducted with accelerometer-assessed mean minutes of weekday MVPA at T1 as the candidate primary outcome in a definitive trial. The model included covariates for intervention arm, local authority, recruitment strategy and mean minutes of weekday MVPA at baseline (T0) and accommodated variation in outcome between schools using a random effect. The difference in mean weekday MVPA was presented with its 95% confidence interval. This analysis was repeated with stratification by sex and for the other accelerometer-derived secondary outcomes.

We conducted a post hoc comparison of control and intervention schools in the amount of physical activity during the after-school period (3 to 5 p.m.) using an adaptation of the above statistical model to estimate the difference in mean activity with 95% confidence interval. For intervention schools we calculated for each pupil the difference in their physical activity during the after-school period between Action 3:30 club days versus other school days, presenting the mean of these differences with its 95% confidence interval.

### 2.8. Economic Evaluation

A resource checklist adapted from previous studies [11,25] was used to assess the feasibility of collecting cost data. Data were collected on resource use and actual intervention costs incurred by TAs. Cost-effectiveness of Action 3:30 was estimated using T1 MVPA data and Action 3:30-related resources and costs. The cost of Action 3:30 producing an additional minute of weekday MVPA compared with no active intervention was estimated by dividing the cost per pupil by the difference between intervention and control arm weekday minutes of MVPA at T1 (Incremental Cost Effectiveness Ratio). Key contacts at each school were asked to provide a description of each of their current after-school physical activity programmes, including duration of the clubs, cost to the school and cost to parents/guardians, at T0 and T1. These data were used to compare the costs associated with Action 3:30 with existing after-school provision, to explore whether Action 3:30 is an economically viable option for primary schools to consider.

### 2.9. Progression Criteria for Conducting a Definitive Trial

The following five criteria were agreed with the funding agency as progression criteria to full-trial:

(a) 25% of schools that are approached agree to join the study
(b) 25% of eligible Year 4/5 pupils express an interest in the study by returning consent forms.
(c) At least 40% of participants expressing an interest in the study are girls
d) At least 50% of the participants in the intervention arm attend 50% of the sessions
(e) At T1, at least a small benefit for weekday MVPA is observed for boys & girls, comparing intervention to control schools, and the upper bound of the 95% CI for each difference exceeds a 10-minute benefit for the intervention group.

## 3. Results

### 3.1. School Recruitment

**Figure 1:**
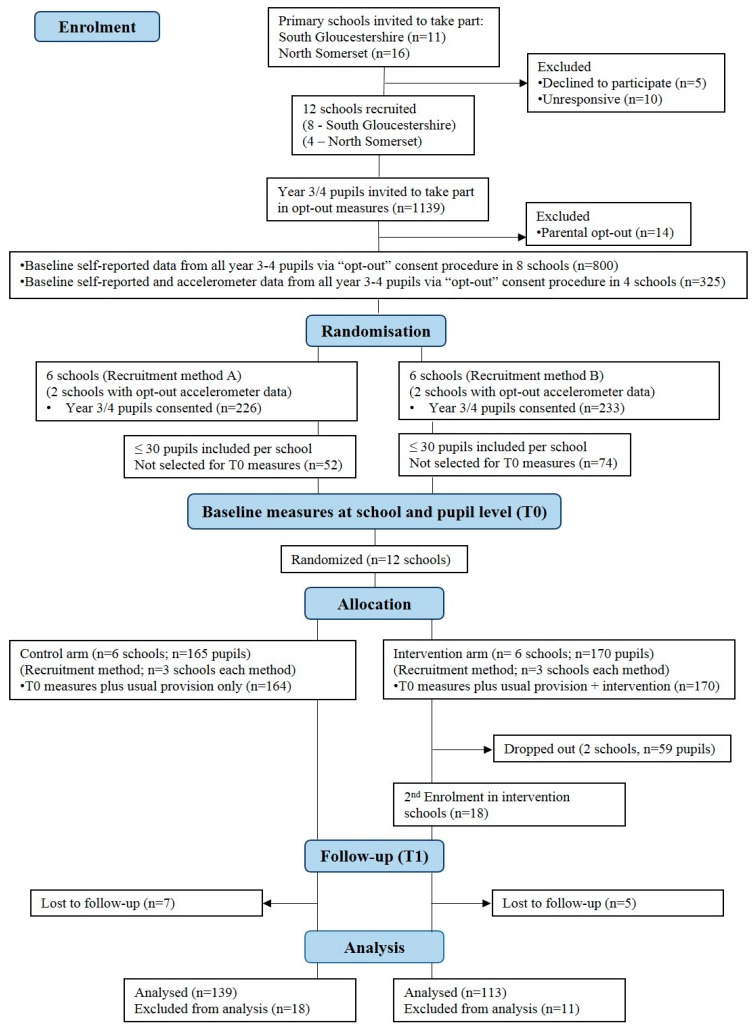
Flow diagram for Action 3:30R trial (based on CONSORT 2010 flow diagram).

A total of 44% of schools that were approached agreed to take part in the study, meeting progression criteria a. Figure 1 presents a flow diagram for the study. An aim of the recruitment process was to recruit half of the schools in each local authority from above the median percent for free school meals (FSM %) of that local authority. Six schools in South Gloucestershire (SG) and four schools North Somerset (NS) had more pupils eligible for free school meals (FSM) compared to the average for schools in their respective local authority areas.

### 3.2. Participants and Recruitment

#### 3.2.1. Phase 1: Pre-Baseline (Opt-Out)

Across the 12 schools, 1139 pupils were eligible to participate, and 1125 pupils completed the PAQ-C survey (98.77%) with only 14 pupils’ (1.23%) parents completing opt-out forms to exclude their child from the measures. In four schools, pupils were also asked to wear an accelerometer for seven days. The percentage of pupils recruited was similar when pupils were asked to wear an accelerometer for 7 days and complete the PAQ-C survey (99.01%) compared to when asked to only complete the survey (98.19%) suggesting this measure and consent process was acceptable to pupils and their parents.

#### 3.2.2. Baseline (T0)

A total of 459 pupils returned parental consent forms (41.39% of eligible), 228 (48.66%) of whom were girls, meeting progression criteria b and c. The number and percentage of pupils recruited was similar for standard (226/557, 40.57%, 6 schools) and enhanced (233/582, 40.03%, 6 schools) recruitment methods. There was no meaningful difference in the PAQ-C scores between standard recruitment (mean 3.2, standard deviation 0.12) and enhanced recruitment (mean 3.08, standard deviation 0.14) or the proportion of girls recruited between standard recruitment (51.32%) or enhanced recruitment (48.07%) (Figure 1). Thus, there were no differences between recruitment methods.

To determine the effectiveness of recruiting low active children, accelerometer and PAQ-C data were used to compare physical activity levels of those who did and did not consent to participate in the intervention. In the four schools with pre-baseline accelerometer data, mean weekday minutes of accelerometer-measured MVPA was 63.23 (SD 19.69) versus 66.75 (SD 18.77) for non-consenting versus consenting participants respectively. Across all 12 schools, PAQ-C scores were 3.13 (SD 0.71) versus 3.21 (SD 0.68) respectively for those who did and did not consent to participate. Minimal differences between groups suggested that the study appealed to children across the physical activity spectrum and that the study was able to recruit high and low-active children within schools.

A total of 335 pupils provided data at T0 with 165 pupils at schools randomly allocated to the control group and 170 at schools randomly allocated to the intervention group (see Figure 1). Following randomisation into trial arms, two schools that were randomised to the intervention group withdrew from the study. In one school this was due to a lack of staff to deliver the intervention. The other school withdrew from the project after a change in the school leadership and did not communicate any further. These two schools were therefore dropped from the primary analyses to obtain an indication of the impact of the intervention in schools able to deliver it. Recruitment and data provision figures for T0 are presented in Supplementary Table S1. Control and intervention groups did not differ by demographics (Supplementary Table S2), physical activity or psychosocial outcomes (Supplementary Table S3) at T0.

### 3.4. Follow up (T1) Comparison between Trial Arms

After adjustment for local authority, recruitment strategy and baseline MVPA, mean weekday MVPA at T1 was very similar between control and intervention groups, overall and for boys and girls separately (Table 1).

Table 1 presents a comparison of the primary and secondary physical activity outcomes between trial arms. Confidence intervals spanning zero suggest no meaningful difference between groups for primary or secondary PA outcomes. Likewise, the proportion of pupils meeting the 60 min MVPA per weekday guidelines appeared to be no different in the intervention group compared to the control group overall and among the boys and girls separately. The proportion of boys who met the guidelines in both intervention and control groups was generally higher than the proportion of girls who met the guidelines.

Table 2 presents exploratory post hoc analyses of physical activity in the after-school period (3 to 5 p.m.) on weekdays. No difference between groups was observed at T0. Comparing intervention and control schools across all weekdays at T1 there is evidence of greater MVPA and of lower sedentary time in the intervention group. Comparing Action 3:30 days to other school days in the intervention group, there is strong evidence of greater MVPA, counts per minute, and LPA, and of a lower sedentary time on Action 3:30 days.

Exploration of psychosocial and BMI variables also found no meaningful difference between trial arms at T1 in the adjusted models (Table 3).

The number of active travel days from school and number of after-school clubs attended (excluding Action 3:30) was slightly lower in the intervention group versus control (2.21 vs. 2.63 and 2.31 vs. 2.49 respectively), however 95% confidence intervals spanning zero suggest the differences are not meaningful. There were no differences in the psychosocial variables.

### 3.5. Economic Evaluation

#### 3.5.1. Action 3:30 Costs

The total cost of the Action 3:30 intervention across the four intervention schools, including training, preparation and delivery costs was £7422.15, with an average cost per school of £1855.55 (based on 2017–2018 prices) (Table 4). Delivery of the intervention after one year was estimated by excluding the training costs, which reduced the total cost to £5929.73 and the average cost per school to £1482.44. The cost of Action 3:30 per pupil, based on a class of 30 attending 30 1-h sessions was estimated to be £61.85 (95% CI £55.44, £68.26), equivalent to £2.06 (95% CI 1.85, 2.28) per pupil per individual session. These estimated costs were reduced to £49.41 (95% CI 43.00, 55.83) and £1.64 (95% CI 1.43, 1.86), respectively after one year, when training costs were excluded. As this was a feasibility trial and not powered to detect significant differences in MVPA, there was no firm basis for estimating cost-effectiveness, however a full break down of the Action 3:30 resources and costs has been produced.

#### 3.5.2. Health-Related Quality of Life

KIDSCREEN-10 and CHU9D responses were analysed to explore health-related quality of life and are presented in Supplementary Tables S4 and S5. As responses to the KIDSCREEN-10 and CHU9D were found to be negatively skewed, median derived T-scores and utility values, respectively, were used in analysis. Mean KIDSCREEN-10 T-scores fell within the normative range of KIDSCREEN-10 European and UK T-scores for children aged 8-11 years [20]. Findings showed no difference in T-scores or utility values between intervention and control group at baseline (T0) or T1, suggesting no difference in health-related quality of life.

#### 3.5.3. After-School Physical Activity Provision

Data from key contacts in each school showed that on average, intervention schools provided more after-school physical activity clubs per week at baseline (T0) and at T1 when compared to control schools. In addition, delivery of after-school clubs cost more in intervention schools compared to control schools at T0 and T1 (see Table 5). The total average cost to schools per pupil per one 1-h session of existing after-school physical activity club at T1 was £5.91 (SD = 13.02). This cost is much higher than the estimated cost of £1.64 for the Action 3:30 club, which suggests that Action 3:30 may provide an economically viable alternative for schools. However, a definitive trial, would be required to confirm this.

## 4. Discussion

The aim of this study was to explore the feasibility and potential efficacy of a revised teaching assistant-led intervention designed to increase levels of weekday MVPA in boys and girls. Five criteria were agreed with the funder for progressing to a definitive trial, as listed in the Methods. Criteria a–d were met; 44% of schools approached joined the study, 43% of eligible pupils expressed an interesting in participating, of which 49% were girls and 70% of pupils attended at least half of the 30 sessions, with attendance being similar between boys and girls. The fifth progression criterion (e) focused on the evidence of promise for an increase in weekday MVPA among boys and girls in the intervention group compared to the control group at follow-up. Our data showed that the pupils in intervention and control groups engaged in similar levels of weekday MVPA at the end of the intervention, suggesting that Action 3:30 replaced existing PA rather than providing additional PA. These findings indicate that the Action 3:30 intervention is feasible to deliver, however there was no evidence that it increased overall MVPA levels in the intervention group.

Exploratory, post-hoc examination of PA levels in the window immediately after school (3–5 p.m.) revealed that the intervention was effective at increasing MVPA straight after school on days when children attended the Action 3:30 club (Table 2). A difference in MVPA of 9 min per day between Action 3:30 days and non-Action 3:30 days in the intervention group suggests that, even though children may have attended other after-school clubs on other days, when they attended Action 3:30 they were more active between 3–5 p.m. than on other weekdays. This echoes findings from a previous intervention study targeting after-school PA [25] which showed that when after-school programs are added to existing provision they increase physical activity on those days but the dose is not sufficient to impact on average physical activity across the week. This finding therefore provides support to Beets and colleagues’ Theory of Expanded, Extended and Enhanced Opportunities (TEO) [26]. TEO hypothesises that increasing the number, duration and quality of PA opportunities for children will lead to increases in PA. Action 3:30 delivers on both expansion (number of opportunities) and enhancement (quality of opportunities) but programs need to be delivered more often to have a sustained impact on average physical activity. Findings therefore indicate a need to increase the provision of high-quality physical activity opportunities after-school.

We have shown in this study and in our previous work [11] that training TAs to deliver an after-school physical activity programme is feasible within primary schools. Offering TAs training to be able to deliver physical activity provision aligns well with government [27] and Ofsted [28] recommendations and is therefore appealing to schools as well as staff, as reflected in the recruitment data. The recent doubling of the PE and Sport Premium allows primary schools to invest more money into continuous professional development for their staff; something that schools are now doing [29] and which adoption of Action 3:30 could facilitate. It is important to note, however, that two schools randomly allocated to the intervention in this study were unable to deliver the intervention for unforeseen reasons, highlighting that individual school factors can act as a barrier to adoption of opportunities such as Action 3:30 regardless of intention. More engagement may have been needed at the recruitment stage to understand school structure and staff capacities in the two schools that could not deliver.

Primary schools often choose to spend their PE and Sport Premium funding on paying for extra-curricular physical activity provision [29]. Our findings showed that on average schools in this study spent £2.36 ± 7.85 at baseline and £5.91 ± 13.02 at follow-up per child per after-school physical activity club session, which is comparable to other primary schools in the UK [12]. The cost of Action 3:30 per session compares favourably, as the estimated cost of one Action 3:30 session per pupil was £2.06, which would be reduced after one year to £1.64 (this excludes the one-off training cost). There is a lack of reporting on the costs associated with children’s physical activity interventions, however the estimated costs of Action 3:30 are comparable to those that have been reported in recent years [25,30]. Primary schools may wish to consider the Action 3:30 programme as an inexpensive alternative to their current provision which is likely to yield similar levels of physical activity and convey the added benefit of contributing to staff development.

Our economic evaluation also revealed that intervention schools provided slightly more and spent more money on after-school physical activity clubs than control schools in this study. The results from our process evaluation (under review) also found that some pupils did not attend all the Action 3:30 sessions because they chose to do other clubs or activities instead. Together this evidence may suggest that because intervention schools already provided a variety of after-school provision, pupils were likely to switch between clubs, which has been similarly found in other PA interventions [11,31]. Therefore, any intervention effect is likely to be attenuated.

Many previous PA interventions in children have not reported the representativeness of participants [32]. An objective of this feasibility study was to recruit a range of pupils including those who have low physical activity levels. To assess the representativeness of our sample, we conducted a pre-baseline survey involving all eligible children in each school. Our results showed minimal differences in self-reported and objectively measured physical activity between those who consented to join the study and those who did not, which is in line with previous literature [33,34,35]. We also showed at baseline that 40% and 38% of control and intervention pupils respectively did not meet the recommended 60 min of MVPA per day, which suggests that we were able to engage a range of pupils across the PA spectrum, including the low active children. However, because the majority of pupils did meet the guidelines it supports the theory that many children already took part in after-school clubs which were swapped for Action 3:30.

Many studies have shown that girls are less active than boys [36,37]. Therefore, PA interventions in children should consider ways to engage girls, to help tackle the age-related decline, which occurs at an earlier age in girls [38]. Based on the findings from the previous Action 3:30 study [11] we revised the intervention to appeal more to girls, and 49% of those who consented to join this study were girls. Additionally, attendance by girls was high. On average girls attended 19 of the total 30 sessions, which was comparable to the boy’s average attendance (20 sessions). Our evidence suggests that Action 3:30 could be recommended to primary schools as a programme which appeals to and engages girls as well as boys in physical activity.

In this study we found that there was no evidence that any of the motivation or health related quality of life variables of the participants in the intervention group were different to the control group at the end of the study. As there was also no evidence of an overall impact on physical activity it is unclear if this a function of the measurement tools, i.e. they were unable to detect differences or merely attributable to the overall lack of impact. It is no possible to untangle these issues without first detecting an intervention effect. As such, there is a need for further examination of the utility of these measures in childhood studies.

A major strength of this study was the rigorous design, with clear research questions that mapped onto five progression criteria. Both low and high deprived schools were recruited, and pupil recruitment rates were relatively high. An opt-out phase was used to assess representativeness of the study population and a re-enrolment point was introduced half-way through the intervention, both of which increased external validity of the study. Data provision rates were high at baseline and follow-up for all measures and an objective measure of physical activity (accelerometers worn for seven days) was used. The main limitation of the study was that two schools were unable to deliver the intervention, one of which did not provide a reason for withdrawal. Generalisability is also limited due to the small number of schools involved, however this is inherent in the design of a feasibility trial.

## 5. Conclusions

While feasible to deliver, no evidence was found that the Action 3:30R intervention increased physical activity compared with a control group, potentially because pupils were swapping one club for another instead of adding new activities. The revised programme appealed to and engaged children across the physical activity spectrum, including girls, and was cheaper to deliver than existing provision with the added benefit of developing staff—something which aligns with current school objectives. Providing activities in the after-school period is important for helping children to meet the physical activity recommendations [10]. Work from the US has suggested focusing on improving the quality of existing after-school provision [26,39], and it may be the case that there is a need to evaluate enhancements to existing provision in addition to creating new provision to improve the quality of after-school physical activity programmes in the UK.

## Figures and Tables

**Table 1:** Physical activity and sedentary time at T1 by trial arm.

Variable	Control			Intervention			
	*N*^a^	Mean	SD	*N*^a^	Mean	SD	I vs. C Adjusted Difference in Means (95% CI) ^b^
**Primary outcomes**							
Weekday MVPA mins	139	58.28	19.72	113	58.33	19.28	−0.5 (−4.57, 3.57)
Boys’ weekday MVPA mins	65	64.06	22.60	50	65.37	20.26	0.06 (−6.59, 6.72)
Girls’ weekday MVPA mins	74	53.20	15.22	63	52.74	16.60	−0.79 (−5.65, 4.07)
**Secondary outcomes**							
Overall mean mins of MVPA	139	55.41	18.99	113	54.53	17.45	−0.75 (−4.49, 3.00)
Mean weekday sedentary mins	139	474.57	60.04	113	481.61	63.96	10.01 (−6.3, 26.31)

^a^ *N* = total pupils providing ≥3 days of valid accelerometer data, by group; ^b^ I vs. C adjusted for local authority, recruitment strategy and baseline MVPA and school clustering.

**Table 2:** Post hoc exploration of mean (SD) PA and sedentary time after-school between 3–5 p.m.

	TO ^a^		T1 ^b^ (all)				T1 (intervention only)			
Status	Con ^c^	Int ^d^	Con ^c^	Int ^d^			Non-Club	Club		
					diff	95% CI			diff	95% CI
N	151	157	139	113			113	111		
MVPA ^e^ mins	12.63 (6.66)	12.01 (6.01)	11.96 (5.71)	13.66 (5.85)	1.70	0.26, 3.14	10.38 (9.26)	18.99 (9.01)	8.62	6.95, 10.29
LPA ^f^ mins	40.29 (8.41)	39.80 (8.39)	38.20 (7.36)	38.44 (7.77)	0.25	−1.63, 2.13	36.38 (8.96)	42.20 (9.45)	5.82	3.96, 7.68
CPM ^g^	787.43 (447.71)	740.50 (323.70)	692.10 (294.10)	755.88 (269.89)	63.82	−6.89, 134.54	639.60 (297.41)	981.97 (409.40)	342.37	267.63, 417.11
SED ^h^ mins	62.50 (11.72)	63.45 (12.18)	66.70 (10.86)	63.97 (11.01)	−2.73	−5.46, −0.01	69.52 (12.48)	55.91 (14.99)	−13.61	−16.72, −10.50

^a^ T0 = baseline, ^b^ T1 = follow up (end of the intervention period); ^c^ Con= control group; ^d^ Int = intervention group; ^e^ MVPA = moderate to vigorous physical activity; ^f^ LPA = light intensity physical activity; ^g^ CPM = average counts per minute across wear period; ^h^ SED = sedentary.

**Table 3:** BMI and questionnaire-derived secondary outcomes at T1.

Variable	Control		Intervention		I vs. C Difference in Means (95% CI) ^c^
	Mean	SD	Mean	SD	
BMI ^a^	17.67	2.61	17.61	2.87	0.17 (−0.15, 0.50)
zBMI ^a^	0.46	1.09	0.32	1.16	0.02 (−0.13, 0.17)
Autonomous motivation for PA	3.42	0.64	3.46	0.58	0.06 (−0.08, 0.29)
Controlled motivation for PA	1.44	0.82	1.57	1.00	0.08 (−0.12, 0.29)
Autonomy need satisfaction	4.92	0.86	4.95	0.85	0.03 (−0.17, 0.22)
Competence need satisfaction	4.75	0.88	4.81	0.76	−0.02 (−0.21, 0.16)
Relatedness need satisfaction	4.96	0.96	5.07	1.00	0.06 (−0.17, 0.29)
Self-esteem	3.69	0.50	3.76	0.50	−0.01 (−0.13, 0.11)
Peer support	18.00	3.81	19.00	4.22	0.74 (−0.49, 1.97)
Number of active travel days to school ^b^	2.50	2.29	1.80	2.24	0.03 (−0.49, 0.30)
Number of active travel days from school ^b^	2.35	2.27	1.94	2.24	0.20 (−0.64, 0.24)
Number of after-school clubs attended	1.70	1.38	1.44	1.28	−0.11 (−0.42, 0.21)

^a^ Body measurement data; ^b^ Based on number of days walked or cycled to and from school; ^c^ I vs. C adjusted for local authority, recruitment strategy and baseline MVPA and school.

**Table 4:** Action 3:30 costs and resources.

Category and Description of Resources	Unit cost (*£*)	Number of Units	Total Cost (*£*)	Mean (*SD*) Cost (*£*) per School	Mean Cost (£) per Pupil ^d^
**Recruitment and marketing costs ^a^**	-	-	3560.67	-	-
**One-off training resources**					
Lead instructor induction training of TAs	-	-	750.00	187.50	6.25
Venue hire for induction training of TAs	-	-	300.00	75.00	2.50
Teaching cover to release TAs for training	-	-	442.42	110.61	3.69
Sub-total			1494.42	373.11	12.44
**Recurrent programme preparation resources**					
Printing: Training guide	18.85/guide	9 guides	169.65	42.41	1.41
Printing: Delivery manual for TAs	16.02/manual	9 manuals	144.18	36.05	1.20
Sports equipment	200.00	4	800.00	200.00	6.67
Sub-total			1113.83	278.46	9.28
**Recurrent programme delivery resources**					
Programme delivery ^b^		240 h	3828.30	957.08 (120.89)	31.90
Lead instructor email/phone support of TAs	25/hour	24 h	600.00	150.00	5.00
Printing materials for programme delivery ^c^	0.17/activity card	2280	387.60	96.90	3.23
Sub-total			4815.90	1203.98	40.13
Indicative total cost			7422.15	1855.55 (120.89)	61.85 (95% CI 55.44, 68.26)
Mainstream indicative total cost after one year (excluding one-off training)			5929.73	1482.44 (120.89)	49.41 (95% CI 43.00, 55.83)
Total cost per pupil per session ^e^					2.06 (95% CI 1.85, 2.28)
Mainstream total cost per pupil per session after one year (excluding one-off training) ^e^					1.64 (95% CI 1.43, 1.86)

^a^ Total cost of recruitment and marketing efforts across all 12 schools. Excluded from indicative total cost. ^b^ Two TAs from each intervention school were paid their existing rate in their school (ranging from £11.28 to £18.00 per hour) to deliver Action 3:30 over 30, 1-h sessions (totaling 240 hours’ delivery time). ^c^ Post-intervention activity cards for intervention group pupils. ^d^ Average cost per school/maximum number of pupils recruited from each school (*N* = 30). ^e^ Average cost per school/maximum number of pupils recruited from each school (*N* = 30)/30 Action 3:30 sessions.

**Table 5:** Summary of T0 and T1 after-school physical activity provision.

Group	Number of Clubs/Week	Club Duration (Mins/Week)	Cost to School ^a^	Cost to Parents ^a^
	Mean (*SD*)	Mean (*SD*)	Mean (*SD*) *£*	Mean (*SD*) *£*
**T0**				
Intervention	3.25 (0.96)	61.15 (14.31)	4.62 (11.27)	2.61 (2.81)
Control	2.83 (1.47)	56.00 (6.87)	0.41 (1.16)	1.57 (1.81)
Total	3.00 (1.25)	58.39 (11.06)	2.36 (7.85)	2.06 (2.34)
**T1**				
Intervention	3.75 (2.06)	65.00 (14.64)	9.00 (15.83)	1.32 (1.62)
Control	3.00 (1.67)	50.28 (7.17)	3.33 (9.85)	1.48 (1.89)
Total	3.30 (1.77)	56.97 (13.28)	5.91 (13.02)	1.41 (1.74)

^a^ Per pupil per club session, based on a 12-week school term.

## Supplementary Materials

The following are available online at http://www.mdpi.com/1660-4601/16/1/131/s1, Table S1: Recruitment and data provision by trial arm at T0; Table S2: Baseline demographics of sample; Table S3: Baseline descriptive statistics of sample; Table S4: Health-related quality of life: baseline and T1 KIDSCREEN-10 Scores; Table S5: Health-related quality of life: baseline and T1 CHU9D Utility values.
