# Supplementary material for: Action 3:30R: Results of a Cluster Randomised Feasibility Study of a Revised Teaching Assistant-Led Extracurricular Physical Activity Intervention for 8 to 10 Year Olds
Source: Int J Environ Res Public Health. 2019 Jan 6;16(1):131. doi: 10.3390/ijerph16010131 (PMC6339197; doi:10.3390/ijerph16010131)
Supplement: Supplementary file 1 [file ijerph-16-00131-s001.pdf]

Supplementary Materials

Action 3:30R: Results of a Cluster Randomised Feasibility Study of a Revised Teaching Assistant-Led Extracurricular Physical Activity Intervention for 8 to 10 Year Olds

Russell Jago [1,4,*], Byron Tibbitts [1], Emily Sanderson [2], Emma L. Bird [3], Alice Porter [1], Chris Metcalfe [2], Jane E. Powell [3], Darren Gillett [5] and Simon J. Sebire [1]

[1] Centre for Exercise, Nutrition & Health Sciences, School for Policy Studies, University of Bristol, Bristol, BS8 1TZ, UK
[2] Bristol Randomised Trials Collaboration, Bristol Trials Centre, University of Bristol, Bristol, BS8 2PS, UK
[3] Centre for Public Health and Wellbeing, University of the West of England, Bristol, BS16 1QY, UK
[4] The National Institute for Health Research Collaboration for Leadership in Applied Health Research and Care West (NIHR CLAHRC West) at University Hospitals Bristol NHS Foundation Trust, Bristol, BS1 2NT, UK
[5] Bristol City Council – Sport & Healthy Lifestyles Team, Healthy Lifestyles Healthy Place, Public Health City Hall, Bristol, Bs3 9FS, UK

Supplementary Table S1: Recruitment and data provision by trial arm at T0.

School	Trial arm *	Eligible	Provided consent (n, % of eligible)				Enrolled (*n*, % of consenting)				
			n	%	Female	%	n	%	CM (n)	PQ (n)	Acc (n)
21	C	131	61	46.6	31	50.8	30	49.2	29	25	28
22	C	112	38	33.9	18	47.4	30	78.9	29	29	24
23	C	171	70	40.9	36	51.4	30	42.9	30	29	30
26	C	58	27	46.6	14	51.9	27	100.0	27	25	25
31	C	57	22	38.6	8	36.4	22	100.0	22	20	20
34	C	54	26	48.1	14	53.8	26	100.0	26	20	24
**All**	**C**	**583**	**244**	**41.9**	**121**	**53.8**	**165**	**67.6**	**163**	**148**	**151**
24	I	78	28	35.9	13	46.4	28	100.0	28	26	23
25	I	48	19	39.6	8	42.1	19	100.0	19	12	18
27	I	108	40	37.0	20	50.0	30	75.0	30	30	28
28	I	113	31	27.4	14	42.4	31	100.0	31	31	31
32	I	53	32	60.4	18	56.3	32	100.0	32	30	29
33	I	156	65	41.7	34	52.3	30	46.2	30	29	29
**All**	**I**	**556**	**215**	**38.7**	**107**	**52.3**	**170**	**79.1**	**170**	**158**	**158**
**Total**	**n/a**	**1139**	**459**	**40.3**	**228**	**52.3**	**335**	**73.0**	**333**	**306**	**309**

* C=control I=intervention; CM = Child measures completed (questionnaire and physical); PQ = Parent questionnaire completed; Acc = Provided ≥3 valid days of accelerometer data (valid day ≥500 minutes wear time)

Supplementary Table S2. Baseline demographics of sample.

	Control (n=165)		Intervention (n=170)	
	**n**	**% of sample**	**n**	**% of sample**
Female	83	50.30	86	50.59
**Parent education:**				
up to GCSEs/GCEs/O levels or similar	27	18.24	27	17.20
A levels/NVQs/GNVQs	53	35.81	45	28.66
first degree/ diploma/HNC/HND	54	36.49	68	43.31
higher degree (e.g. MSc/PhD)	14	9.46	17	10.83

Supplementary Table S3. Baseline descriptive statistics of sample.

	Control			Intervention		
	**n**	**Mean**	**SD**	**n**	**Mean**	**SD**
Age *	151	8.4	0.63	158	8.35	0.68
BMI (weight/height^2)	165	17.21	2.35	170	16.86	2.46
zBMI	165	0.47	0.99	170	0.26	1.15
IMD score	152	16.11	9.10	159	14.10	12.08
Total PA [a]	151	648.27	211.93	157	625.62	177.49
Mean overall MVPA mins	151	66.06	21.27	157	65.43	20.94
Mean weekday MVPA mins	151	65.75	22.45	157	64.96	21.88
Mean overall Sedentary mins	151	448.39	52.07	157	456.17	60.79
Mean weekday Sedentary mins	151	457.23	57.09	157	465.81	60.86
Autonomous motivation PA	165	3.41	0.69	170	3.35	0.68
Controlled motivation PA	163	1.71	0.89	170	1.68	0.94
Autonomy need satisfaction	165	4.83	0.89	170	4.70	0.96
Competence need satisfaction	165	4.69	0.98	170	4.72	0.86
Relatedness need satisfaction	165	5.03	0.99	170	5.02	0.92
**School Travel Mode:**						
Number of active travel days to school *[b]	154	2.71	2.27	163	2.44	2.30
Number of active travel days from school *[b]	154	2.55	2.28	163	2.84	2.24
Number of after-school clubs attended *	152	1.84	1.41	160	1.56	1.45

[a] Mean accelerometer counts per minute across all valid days; [b] Based on the average reported number of days walked or cycled to and from school; * Derived from parental questionnaire; IMD = index of multiple deprivation score (higher = less affluent); PA = physical activity; MVPA = moderate to vigorous physical activity; BMI = body mass index, zBMI = age- and sex-adjusted score compared with UK reference values [38]

Supplementary Table S4. Health-related quality of life: baseline and T1 KIDSCREEN-10 Scores.

**Outcomes**[a]	*N*	**Mean *T*-score**	*SD*	**Median *T*-score**	**IQR (25th quartile)**	**IQR (75th quartile)**
**Baseline**						
Intervention	106	50.28	12.08	48.29	41.24	57.29
Control	163	50.51	10.21	49.76	43.35	55.07
Total	269	50.42	10.96	48.29	42.81	55.07
**T1**						
Intervention	105	51.56	11.76	49.76	42.27	57.29
Control	163	49.31	10.39	46.94	42.27	53.11
Total	268	50.19	10.98	48.29	42.27	55.07

[a] Higher scores indicate a better health-related quality of life. European normative mean *T*-scores for children aged 8-11 years range from 48.53 to 59.27. UK normative *T*-scores for children aged 8-11 years range from 45.40 to 54.66.

Supplementary Table S5. Health-related quality of life: baseline and T1 CHU9D Utility values.

Outcomes[a]	*N*	Mean utility value	*SD*	Median utility value	IQR (25th quartile)	IQR (75th quartile)
**Baseline**						
Intervention	106	0.86	0.11	0.88	0.79	0.94
Control	163	0.88	0.10	0.88	0.81	0.95
Total	269	0.87	0.10	0.88	0.81	0.95
**T1**						
Intervention	106	0.85	0.11	0.86	0.77	0.94
Control	163	0.85	0.10	0.87	0.79	0.92
Total	269	0.85	0.11	0.86	0.79	0.92

[a]A higher value indicates better health-related quality of life. CHU9D utility scores range from 0.00 (equivalent to being dead) to 1.00 (perfect health).
